# Type I bare lymphocyte syndrome with novel TAP1 and TAP2 pathogenic variants

**DOI:** 10.1016/j.jdcr.2024.05.042

**Published:** 2024-06-25

**Authors:** Sahal Samarkandy, Randa Khafaji, Alhusain Alshareef

**Affiliations:** a Division of Dermatology, Department of Medicine, King Abdulaziz Medical City, Jeddah, Saudi Arabia; b Medical Genomics Research Department, King Abdullah International Medical Research Center, Ministry of National Guard - Health Affairs, Jeddah, Saudi Arabia; c Medicine Department, King Abdullah Medical Complex, Jeddah, Saudi Arabia

**Keywords:** bare lymphocyte syndrome, bare lymphocyte syndrome type I, HLA class I deficiency

## Introduction

Type 1 bare lymphocyte syndrome (type I BLS) is a rare recessive immunogenetic disorder characterized by disruption in the expression of human leukocyte antigen class I, also known as major histocompatibility complex class I.^1^ The intricate process of loading intracellularly processed antigens onto human leukocyte antigen/major histocompatibility complex class I molecules is imperative for their maturation and subsequent stable presentation on the cell surface.^1^^,^^2^ Hence, genetic mutations impacting the mediator complex responsible for this loading phase, namely the transporter associated with antigen processing (TAP) complex, lead to the manifestation of type I BLS.^1^^,^^2^ Here, we report a case of type I BLS in Saudi Arabia with novel pathogenic variants of TAP1 and TAP2 genes.

## Case report

**Fig 1:**
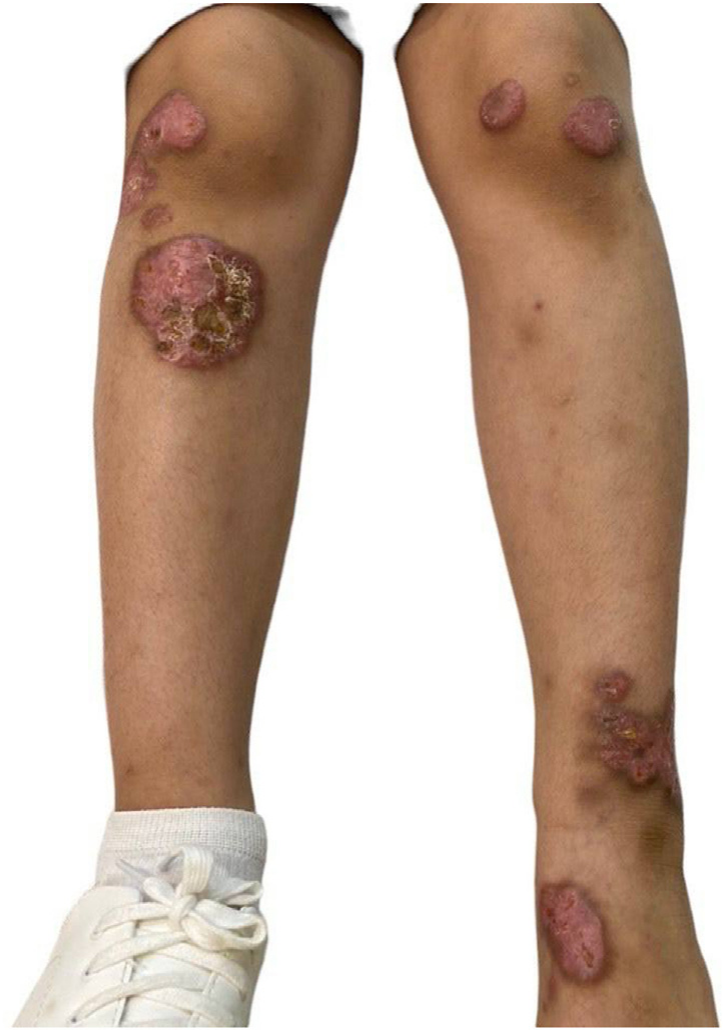
Six large well-demarcated round indurated plaques with some areas of ulcerations and yellowish crusts distributed asymmetrically over both lower extremities.

**Fig 2:**
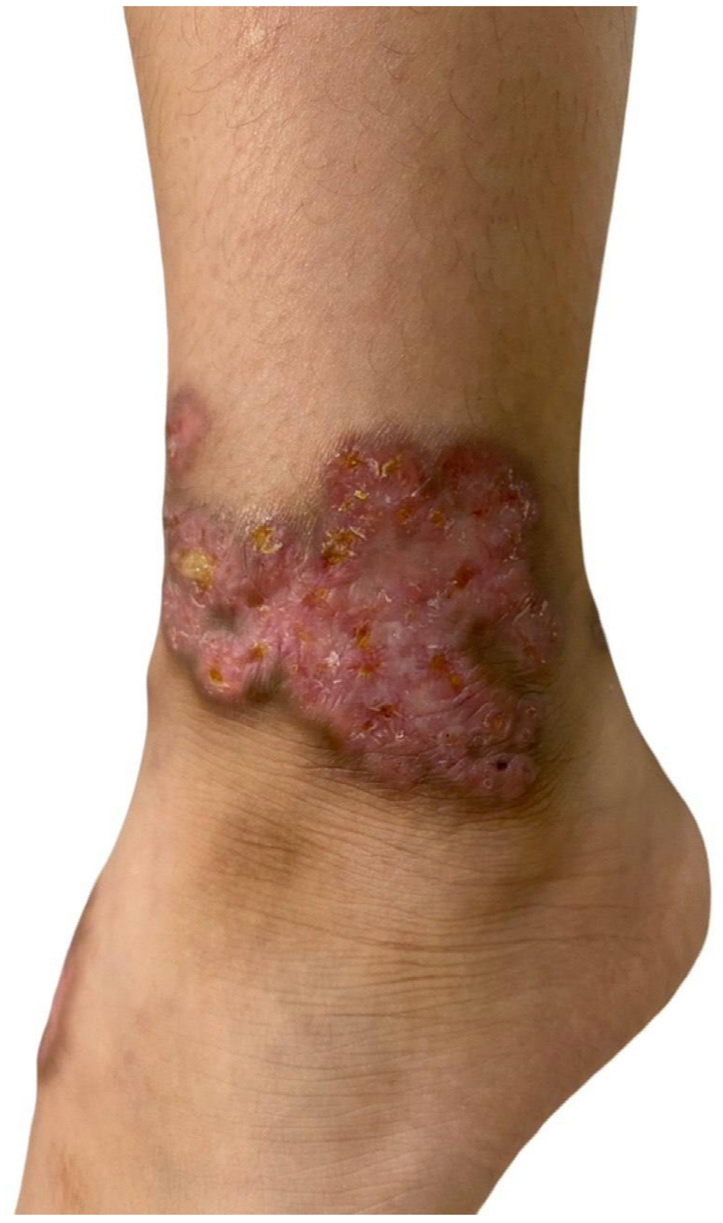
Multiple well-demarcated erythematous ulcerated plaques coalescing over the left ankle.

A 10-year-old girl patient presented with a painless pruritic rash over her lower extremities that started as papules that progressively enlarged. On physical examination, she had 6 large well-demarcated indurated plaques asymmetrically distributed over the lower extremities. The largest lesion, located on the proximal aspect of the right shin, presented an erythematous scaly plaque with an exudative yellowish crust and violaceous borders measuring 6.5 × 6.5 cm in size. Another plaque similar in morphology with a polycyclic configuration was observed on the left ankle (Figs 1 and 2). Her medical history was insignificant aside from a remote history of a resolved reactive airway disease in childhood. Also, she underwent bilateral myringotomy because of recurrent otitis media with effusion.

**Fig 3:**
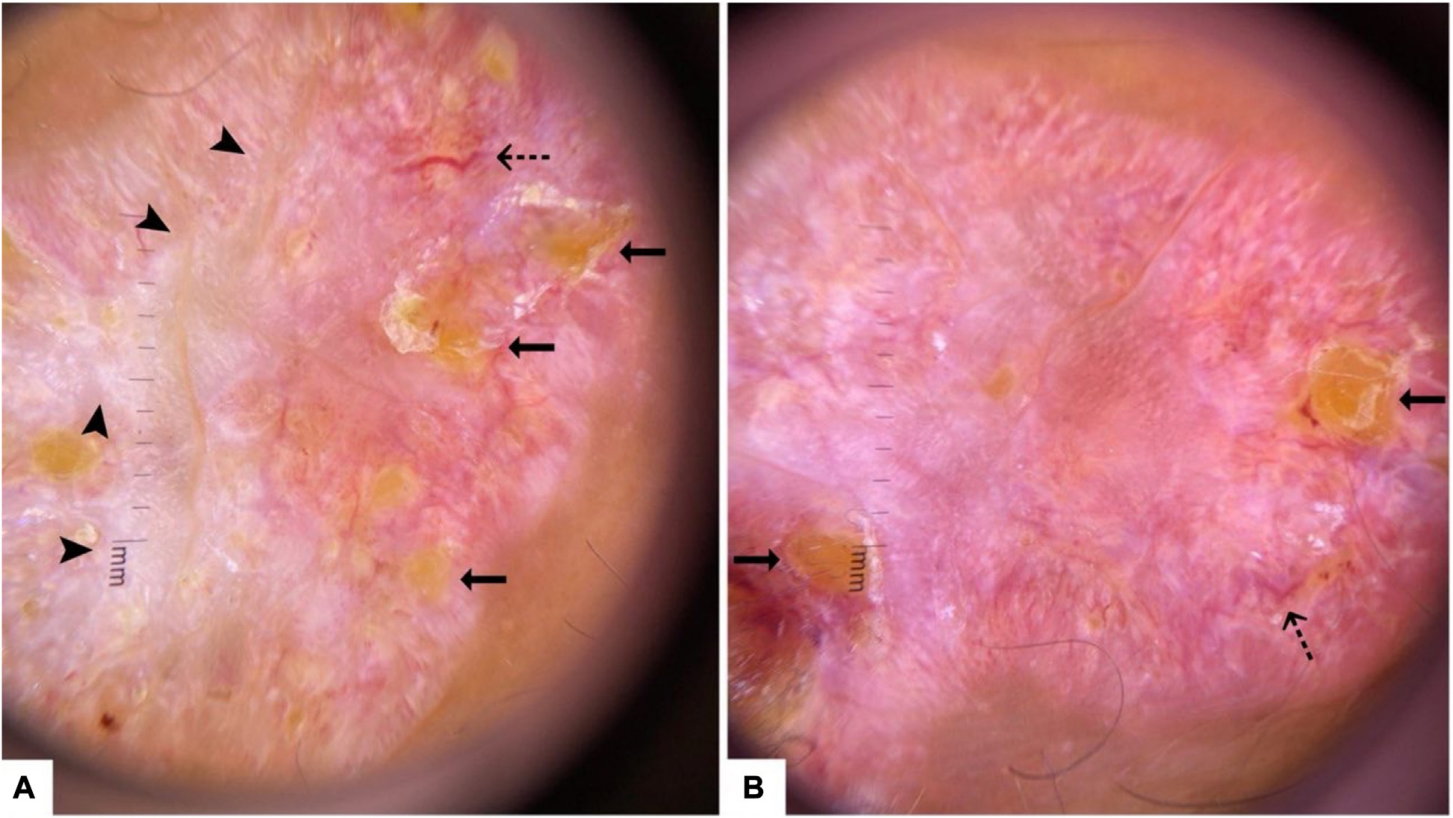
**A, B,** Depicting the dermatoscopic features of the lesions. **A,** Central white scarring (*black arrow heads*), yellow globules (*solid arrows*), and interspersed vessels (*dashed arrows*). **B,** Yellow globules (*solid arrows*) and peripheral telangiectatic vessels (*dashed arrows*).

The dermatoscopic appearance depicted the presence of multiple foci of yellow to orange areas scattered on an erythematous base. Shiny white blotches can be visualized centrally, and interspersed telangiectatic vessels were more notable at the periphery. The findings were consistent with granulomatous inflammatory dermatosis (Fig 3).^3^

A routine laboratory evaluation was performed. The results indicated that the patient had 22.90% lymphocytes with a total white cell count of 12.2 × 10^9^/L. Flow cytometric analysis of blood lymphocyte subsets revealed low T-cytotoxic cells (CD3^+^CD8^+^) number (436.25/mm^3^; normal range, 600-2200 mm^3^), along with normal values of T-helper cells (CD3^+^CD4^+^), B-cells (CD19^+^), and natural killer cells (CD3^–^CD16^+^CD56^+^). Additional immunologic workup showed low levels of immunoglobulin (Ig) M (0.25) and interferon gamma (<0.03 IU/mL); however, normal IgA (0.96), IgE (114.7), IgG (9.13), interleukin 2 (<1.0 pg/mL), and interleukin 10 (<5.0 pg/mL) levels.

**Fig 4:**
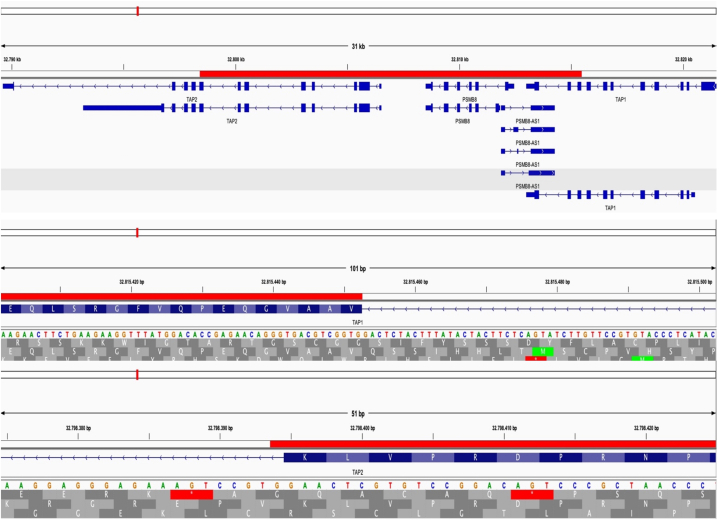
Integrative Genomics Viewer images showcasing the deletions in TAP1 and TAP2 genes.

A 4-mm skin punch biopsy obtained showed focal subepidermal granulomatous inflammation with caseating necrosis. Fungal infections, foreign body reactions, and Leishmania and Mycobacterium infection were excluded by extensive testing. After which, whole exome sequencing identified a pathogenic structural variant confirming the diagnosis of type I BLS (Online Mendelian Inheritance in Man: 604571). The coding exons were enriched using Roche Kapa capture technology and Illumina technology was used for Amplification and sequencing. Identified single nucleotide variants were filtered through external and internal database focusing on rare variants with a minor allele frequency of ≤1% in genome aggregation database. Whole exome sequencing detected a ∼17 kb gene deletion encompassing exons 8 to 11 of TAP1 with the sequence variant ID “NM_000593.5:c.1748_2254_del” and exons 1 to 7 of TAP2 with the sequence variant ID “NM_001290043.1:c.1_1467+1_del” (Fig 4). Specifically, the deletion caused a homozygous variant with the given coordinates, “∼17 Kb deletion chr6:32, 798,394-32,815,452.” To the best of our knowledge, the variant has not been described in literature so far, and allele frequency of this variant in the general population has not been documented.

Treatment with prednisolone 40 mg once daily for 30 days was initiated. Consequently, the granulomatous plaques improved, becoming flatter and smaller in size. Thereafter, prednisolone was tapered down to 20 mg once daily and tacrolimus 1.5 mg twice daily was initiated. On the completion of 60 days on the adjusted regimen, some of the lesions almost completely resolved with hyperpigmented scarring; however, other lesions healed partially.

A clinical decision was made to continue tapering the medications down and switching to thalidomide. The patient was maintained on a dose of 50 mg once daily thalidomide and her condition remained stable even after tapering prednisolone. However, because of eligibility limitations and social reasons the patient discontinued the medication for approximately 18 months and her condition relapsed significantly as her previously healed lesions became extensively ulcerated and coalesced together.

## Discussion

Although type I BLS is extremely rare, with only around 20 cases reported in the literature. This syndrome exhibits significant biological and clinical diversity, notably affecting various genes encoding different components of the TAP complex, such as TAP1, TAP2, or TAPASIN.^3^ The immunologic sequelae of this molecular aberration manifested as a decrease in the number of the CD8^+^ T cells and impaired activity of the natural killer cells.^4^ As observed in the index case, low count of T-cytotoxic cells (CD3^+^CD8^+^) was demonstrated while the remnant immune cells were within normal parameters. However, the low level of IgM and interferon gamma reflects decreased efficacy in the immune mediation properties of these cells.

The hallmark of this syndrome is the presence of sinopulmonary manifestations that tend to start from early childhood as a mild purulent rhinosinusitis and recurrent otitis media with effusion. These manifestations have the propensity to evolve progressively, culminating in recurrent bacterial and parasitic pneumonias, ultimately resulting in respiratory insufficiency. In addition, granulomatous skin ulcers were found in approximately half of the reported cases. Theoretically, this eruption was attributed to an autoimmune process triggered by local infectious agents.^5^ Interestingly and similarly to our patient, only 2 documented cases of type I BLS manifested with progressive ulcerative lesions but lacked sinopulmonary manifestations.^6^^,^^7^

Given the rarity of this disorder, the absence of a definitive cure and standardized management guidelines presents a challenge. However, several treatment modalities have been experimented in the literature with varying responses, including chloroquine, psoralen and ultraviolet therapy, and prednisolone.^7^^,^^8^ Of note, several studies advised against immunosuppressive therapy rendering it ineffective and potentially fatal in cases with concomitant bronchiectasis.^9^ Conversely however, one case has shown that the administration of prednisolone at an initial dose of 40 mg halted the progression of skin lesions for 7 years.^6^ Similarly, the treatment regimen for the case presented included prednisolone as the patient had no pulmonary involvement. Nevertheless, to mitigate the potential long-term adverse effects associated with corticosteroids, the patient was shifted to thalidomide. This medication is known for its inhibitory effects on tumor necrosis factor-alfa and interleukin 12, cytokines recognized for their pivotal roles in sustaining granulomatous inflammation.^10^

To conclude, as type I BLS is a rare disorder with a variety of phenotypic presentations, treatment should be individualized. Notably, early detection and prompt management can not only improve the disease outcome, but also prevent serious consequences and permanent disfigurement.

## Conflicts of interest

None disclosed.
